# Clonal evolution of mycosis fungoides into peripheral T-cell lymphoma: evidence from next-generation sequencing in two cases

**DOI:** 10.1007/s00277-026-07155-1

**Published:** 2026-07-07

**Authors:** Olga Novosad, Alberto Schena, Maria Cristina Pirosa, Helmut Beltraminelli, Luca Mazzucchelli, Emanuele Zucca

**Affiliations:** 1 Clinic of Hematology, Oncology Institute of Southern Switzerland, Ente Ospedaliero Cantonale, Bellinzona, Switzerland; 2 Institute of Oncology Research, Bellinzona, Switzerland; 3 Faculty of Biomedical Sciences, Università della Svizzera Italiana, Lugano, Switzerland; 4 Clinic of Dermatology, Ente Ospedaliero Cantonale, Bellinzona, Switzerland; 5 Clinic of Pathology, Istituto di Diagnostica integrata della Svizzera Italiana, Ente Ospedaliero Cantonale, Locarno, Switzerland

**Keywords:** Peripheral T cell lymphoma, Cutaneous T cell lymphoma, Transformation, NGS

## Abstract

Cutaneous T-cell lymphomas (CTCL) are characterized by marked clinical and molecular heterogeneity. Mycosis fungoides (MF), the most common subtype, usually follows an indolent course but may rarely progress into more aggressive lymphoid neoplasms. Distinguishing between clonal evolution and the development of a second, unrelated lymphoma remains a diagnostic challenge, particularly when conventional T-cell receptor (TCR) gene rearrangement analysis is inconclusive. We report two patients with long-standing, clinically stable MF who subsequently developed rapidly progressive systemic disease with histologically confirmed peripheral T-cell lymphoma (PTCL). In both cases, TCR-gamma PCR analysis suggested distinct clonal populations across different biopsy sites. However, next-generation sequencing (NGS) using a comprehensive lymphoma-targeted gene panel revealed overlapping mutational profiles between skin and lymph node samples, supporting a shared clonal origin. These findings highlight the limitations of PCR-based clonality assessment and demonstrate the added value of NGS in defining clonal relationships and disease evolution in CTCL. Improved molecular characterization may have important implications for diagnosis, classification, and therapeutic decision-making in patients with T-cell lymphomas.

## Introduction

Cutaneous T-cell lymphomas (CTCL) represent a heterogeneous group of non-Hodgkin lymphomas characterized by a variable clinical course and complex molecular background. Mycosis fungoides (MF), the most common subtype, typically follows an indolent course but may, in rare cases, evolve into more aggressive lymphoid neoplasms. Distinguishing between true transformation and the development of a second, unrelated T-cell lymphoma remains a major diagnostic challenge, particularly when conventional clonality assessment using T-cell receptor (TCR) gene rearrangement analysis yields inconclusive or discordant results. Recent advances in next-generation sequencing (NGS) have improved the ability to assess clonal relationships and uncover the molecular evolution of T-cell lymphomas. Here, we report two patients with long-standing MF who developed aggressive peripheral T-cell lymphoma, in whom combined molecular analyses revealed a shared mutational profile, supporting clonal evolution despite discordant PCR-based clonality findings.

## Methods

Histopathological evaluation was performed on formalin-fixed, paraffin-embedded tissue samples obtained from skin and lymph node biopsies. Immunohistochemical staining was carried out using standard protocols with antibodies against CD3, CD4, CD5, CD7, CD8, CD10, BCL6, PD1, CXCL13, TIA1, Granzyme B, CD30, and Ki-67. TCR gamma gene rearrangement analysis was performed by polymerase chain reaction (PCR) on DNA extracted from skin, lymph node, and peripheral blood samples, according to established protocols. NGS was conducted using a custom lymphoma-targeted gene panel including 172 genes (Ente Ospedaliero Cantonale and Universitätsspital Basel) on the Ion Torrent platform (Thermo Fisher Scientific). Library preparation, sequencing, and data analysis were performed according to the manufacturer’s instructions. Variants were classified based on their predicted functional impact and variant allele frequency (VAF).

### Clinical case №1

In January 2023, a 73-year-old woman was referred with widespread lymphadenopathy, fever and severe abdominal pain. She had indolent mycosis fungoides (MF) since 2017, limited to stable skin patches on her right foot (stage IA, T1N0M0B0). Prior therapies included PUVA (2020) and oral acitretin (last dose June 2021). Her only other conditions were well-controlled hypertension and Parkinson’s disease; no other comorbidities were noted.

Initial labs showed mild anemia and moderate neutropenia, with rare, atypical lymphoid cells on the peripheral smear. Bone marrow biopsy and flow cytometry of blood and marrow showed no lymphoma involvement. An 18 F-FDG PET/CT scan demonstrated intensely hypermetabolic enlarged lymph nodes in the retroperitoneum and in cervical and axillary regions (SUVmax 53).

Biopsies were obtained from the persistent skin patch on the toe (the only remaining MF lesion) and from an axillary lymph node. Histology of the toe biopsy confirmed MF without large-cell transformation or CD30 overexpression. The axillary node was effaced by a diffuse infiltrate of small-to-medium atypical T lymphocytes. Immunohistochemistry showed neoplastic T-cells that were CD3+, CD5+, with a high CD4:CD8 ratio (~ 10:1) and partial loss of CD7. The malignant cells lacked markers of follicular helper T-cells (CD10, BCL6, PD1, CXCL13) and of cytotoxic function (TIA1, Granzyme-B); EBV (EBER) was negative. The proliferation index (Ki-67) was ~ 30%. Taken together, these findings were consistent with a peripheral T-cell lymphoma, not otherwise specified (PTCL-NOS) according to the current lymphoma classifications [1, 2].

**Fig. 1:**
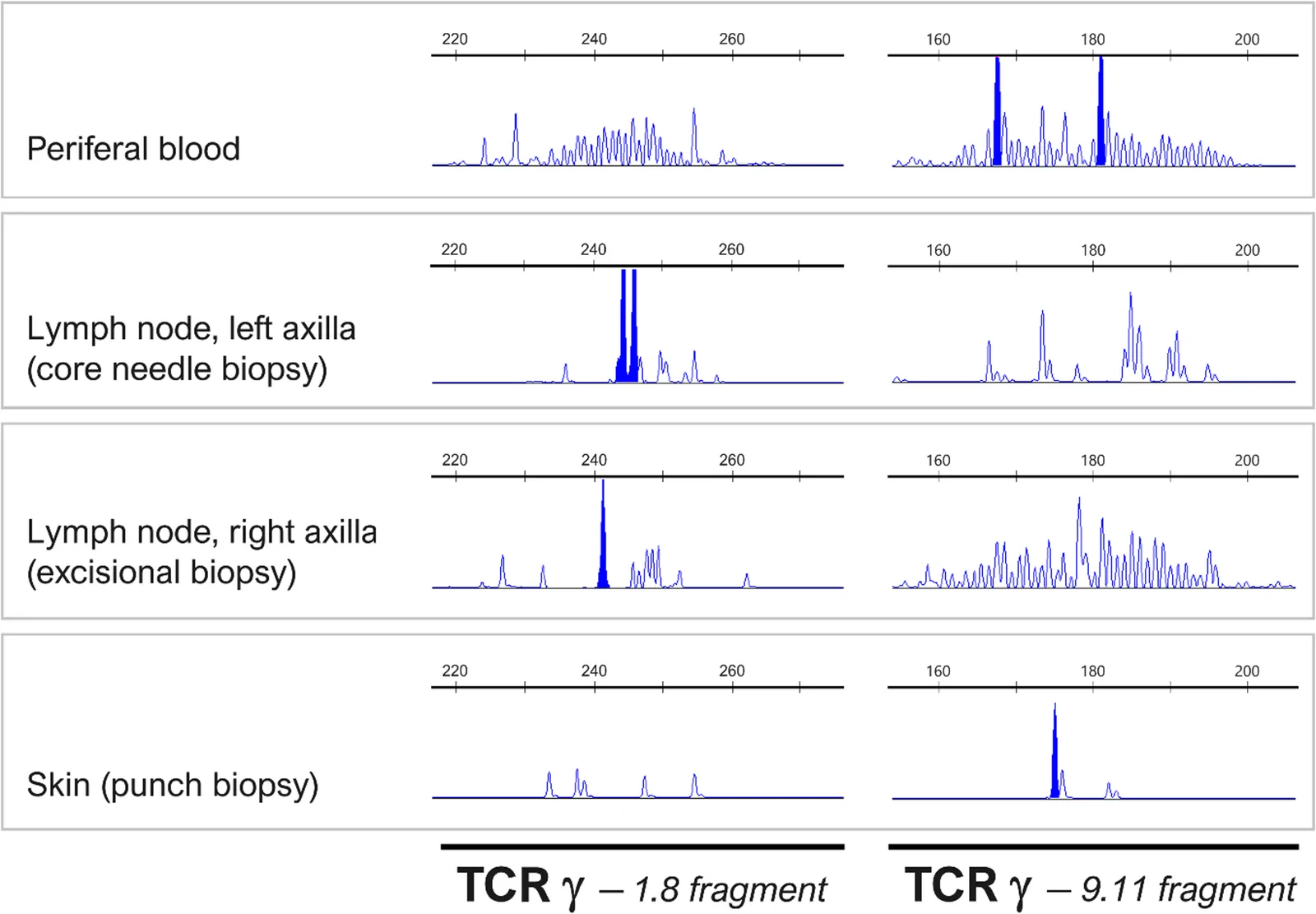
Molecular pathology analyses for TCR-gamma rearrangement in the skin biopsy, lymph node and peripheral blood sample, apparently indicating the existence of clonally unrelated T-cell lymphoproliferative disorders at different anatomical sites

To determine whether there was a clonal relationship between the MF and the lymphoma found in the axillary lymph node, molecular pathology analyses for TCR-gamma receptor rearrangement were performed by Polymerase Chain Reaction (PCR) on the skin biopsy, the lymph node, and on a peripheral blood sample (Fig. 1).

The different profiles suggested the existence of clonally unrelated lymphoproliferative diseases. However, additional next-generation sequencing (NGS)-based multi-gene panel testing (using a custom lymphoma panel containing 172 genes) performed on lymph node and skin biopsy samples demonstrated an overlapping mutational profile, thus indicating a clear clonal relationship, albeit with the acquisition of new mutations in the lymph node (Table 1).

**Table 1:** Case N°1. NGS analysis results on the biopsy tissues from a lymphoma-infiltrated axillary lymph node and from an MF lesion (right foot skin). The same custom multigenic lymphoma panel containing 172 genes was used in both analyses

Biopsy site	Diagnosis	Clonality	Neoplastic cells	NGS results ^a^			
				Gene	Protein variant	VAF^b^	Category ^b^
Right axillar lymph node	Peripheral T-cell lymphomaNOS	TCR-gamma 1–8 fragment monoclonal	70%	SOCS1 SOCS1 EGR2 TRIP12 CIITA FYN PTEN IL16	p.P33Rfs*48 p.A49_P50dup p.S277N p.E829D p.G243Wfs*60 p.N293D p.L108F p.S1048L	6% 33% 62% 47% 14% 16% 16% 8%	F V V V F V V V
Skin (right foot)	Mycosis Fungoides	TCR-gamma 9–11 fragment monoclonal	10–20%	SOCS1 SOCS1 EGR2 TRIP12	p.P33Rfs*48 p.A49_P50dup p.S277N p.E829D	3% 29% 69% 66%	F V V V

^**a**^ Custom Lymphoma Panel (Ente Ospedaliero Cantonale and Universitätsspital Basel), Ion Torrent (Thermofisher)

^**b**^ F(functional): variant that impacts on the protein`s functionality/relevant clinical significance; V: variant of unknown significance; VAF: variant allele frequency

The patient was treated with three cycles of reduced-dose CHOP (cyclophosphamide, doxorubicin, vincristine, prednisone) [3], but an interim PET/CT in June 2023 showed disease progression (Deauville score 5). Given the patient’s age and comorbidities, we decided to offer an alternative second-line therapy with gemcitabine single-drug treatment, which led to a minimal, transient response but also resulted in severe toxicity (thrombocytopenia and neutropenia with recurrent infections and significant fatigue). Alternative agents (belinostat, pralatrexate) were considered but were not available. Unfortunately, the disease progressed to the patient’s death one month after the last specific treatment.

### Clinical case №2

A 79-year-old female patient has a clinical history of CD30-negative mycosis fungoides, diagnosed in 2011, with exclusive cutaneous involvement presenting as plaques and tumors (stage IIB, T3N0M0B0). Over the years, she had undergone various systemic treatments, including interferon and retinoids, with poor tolerance and limited benefit. She had also received radiotherapy on several disease sites, achieving good but temporary control of the lesions. No other significant comorbidities were reported.

In April 2024, the patient presented with fatigue and progressive, exertional dyspnea. An abdominal ultrasound (ordered for mildly elevated liver enzymes) unexpectedly showed a large pre-tamponade pericardial effusion (~ 2.2 cm). Urgent pericardiocentesis drained serous fluid without malignant cells. A thoraco-abdominal angio-CT scan was conducted, revealing a peripheral pulmonary embolism in the lower right lobe artery, for which anticoagulation therapy with edoxaban was initiated. Additionally, bilateral pleural effusions were documented.

After initial improvement, her fatigue and dyspnea worsened over the next weeks, accompanied by new dizziness. In early July 2024, a cardiology evaluation found second-degree atrioventricular block on ECG. Cardiac MRI revealed a 2.5 cm mass on the diaphragmatic wall of the left ventricle, suggesting pericardial tumor involvement. The pericardial and pleural effusions persisted, and small (≈ 1 cm) right axillary lymph nodes were noted.

A biopsy of the enlarged right axillary lymph node confirmed peripheral T-cell lymphoma. Histology showed near-total effacement by medium-sized lymphoid cells with a high mitotic rate (Ki-67 ~ 80%). Immunohistochemical results revealed a neoplastic population of cells expressing CD3, CD5, CD7, and CD8, with partial loss of CD2 expression. CD56, CD30, TdT, and EBER-ISH were negative. As in the previous case, to establish the potential clonal evolution, molecular profiles of the T-cell lymphoma were compared with those of a previous skin biopsy from December 2022, suggestive of MF. Molecular analyses revealed similar TCR gene rearrangements, and an NGS-based multi-gene panel identified a common profile of genetic mutations, suggesting a clonal relationship between the two neoplastic processes (Table 2).

**Table 2:** Case N°2. NGS results on the biopsy tissues from a lymphoma-infiltrated axillary lymph node and from an MF lesion (left arm skin). The same custom multigenic lymphoma panel containing 172 genes was used in both analyses

Biopsy site	Diagnosis	Clonality	Neoplastic cells	NGS results ^a^			
				Gene	Protein variant	VAF^b^	Category ^b^
Right axillar lymph node	PeripheralT-cell lymphoma NOS	TCR-gamma 1–8 fragment monoclonal TCR-gamma 9–11 fragment monoclonal	70%	SPEN SETD2 SETD2 TNFAIP3 TRRAP POT1 KMT2C CDKN2A SETD1B MGA JAK3	p.S1298F p.Q1797 p.R1625C p.K786T p.S722F p.R80H p. ? p.R112H p.P1423S p.T401A p.A573V	44% 35% 27% 49% 39% 14% 10% 37% 33% 60% 45%	V F F V F V V V V V F
Skin (left arm)	Mycosis Fungoides	TCR-gamma 1–8 fragment monoclonal TCR-gamma 9–11 fragment monoclonal	70%	SETD2 SETD2 SPEN TNFAIP3 MGA JAK3 EP300	p.Q1797 p.R1625C p.S1298F p.K786T p.T401 p.V674A p.R1821	37% 31% 34% 55% 44% 27% 5%	F F V V V F F

^**a**^ Custom Lymphoma Panel (Ente Ospedaliero Cantonale and Universitätsspital Basel), Ion Torrent (Thermofisher)

^**b**^ F (functional): variant that impacts on the protein`s functionality/relevant clinical significance; V: variant of unknown significance; VAF: variant allele frequency

A biopsy of the left ventricular myocardium was non-diagnostic, but in context the cardiac findings were interpreted as lymphoma infiltration (a rare but recognized presentation). A staging CT scan showed that the pericardial mass had grown, now extending from the left ventricular wall into the atrium and right ventricle, causing marked cardiac compression. The patient then developed complete (third-degree) heart block requiring a pacemaker and worsening heart failure despite medical therapy. A short steroid trial provided no improvement. Given her condition, she declined further chemotherapy and was maintained on best supportive care.

## Discussion

These two cases represent a complex diagnostic challenge, as they involve distinct T-cell malignancies with partially different molecular profiles.

CTCL comprise a heterogeneous group of non-Hodgkin lymphomas that are generally believed to arise from skin homing memory T cells. However, recent genomic and multiomics studies have revealed substantial genetic heterogeneity in MF, with some cases harboring mutations that appear to precede complete TCR rearrangement. These findings have led to the hypothesis that, at least in a subset of patients, MF may originate from an earlier hematopoietic or T cell precursor that C. The resulting progeny would subsequently differentiate into memory T cells, which constitute the clinically apparent lymphoma [4, 5].

The most common CTCL are MF and Sézary syndrome (SS) [6]. While both share some features, SS typically presents with systemic manifestations such as erythroderma, lymphadenopathy, and circulating cerebriform T cells, and generally carries a poor prognosis. MF is usually more indolent, progressing slowly from patches to plaques and ultimately to tumors. The principal prognostic factor is disease stage, which reflects both skin and extracutaneous involvement. Other unfavorable indicators include the presence of CD30-positive blasts (> 25%) in skin lesions or circulating clonal T cells [1, 2, 6].

Although late-stage MF can evolve into a more disseminated disease resembling or meeting criteria for SS, transformation to aggressive PTCL, is relatively rare and is associated with a significantly worse prognosis. However, the mechanisms underlying transformation are far less well studied than those involved in the progression of indolent B cell lymphomas. Recent multiomics studies in MF have identified upregulation of T cell activation pathways and marked subclonal heterogeneity, suggesting that transformation is driven by monoclonal TCR expansion, metabolic reprogramming, and immune escape mechanisms [7].

The cases discussed here are unusual in that patients with long-standing, stable MF, showed no signs of cutaneous progression, yet subsequently developed aggressive systemic with rapidly growing generalized lymphadenopathy. Initially, a second lymphoma was suspected because of the extracutaneous progression and, particularly in the first case, because TCR rearrangement analyses appeared to show distinct clones across biopsies. However, additional analyses using an NGS panel demonstrated a shared mutational profile, supporting a clonal relationship between neoplastic cells in the skin and lymph nodes.

Tumor subclones in MF can be detected even in early stages of the disease, suggesting that clonal diversity emerges early and supporting the notion that transformed clones retain developmental plasticity [7, 8]. The discordant TCR rearrangements observed in the first patient may therefore reflect divergent clonal evolution arising from a common ancestral clone that acquired oncogenic events prior to T-cell differentiation.

Two main messages emerge from our observations. First, molecular pathology relying solely on conventional TCR polymerase chain reaction (PCR) has important limitations. PCR-based fragment-length analysis can yield false-negative results in early disease and may mislead when assessing clonality between separate biopsies. Identical fragment sizes may represent distinct rearrangements, while somatic mutations acquired during disease progression can alter fragment length, suggesting the presence of subclones rather than two independent lymphomas. These limitations emphasize that TCR PCR results should always be interpreted within the broader clinical and histopathologic context.

NGS-based analyses—either through multigene panels or high-throughput TCR sequencing—allow a more precise assessment of clonal relationships [9–11]. Recent studies have documented marked intratumor genomic heterogeneity in MF [12], likely due to the prolonged natural course of the disease and the emergence of new subclones bearing distinct mutations. The high sensitivity of these assays challenges the traditional notion of clonality based solely on TCR sequence identity or single mutations. As molecular testing becomes more widely available, standardized criteria for interpreting complex molecular results will be essential, especially to differentiate true second lymphomas from sub-clonal evolution within the same neoplasm.

The second point concerns the risk of secondary malignancies in MF. Several studies have reported a modestly increased risk of solid tumors, particularly in the gastrointestinal tract, lung, skin, and urogenital system, whereas the occurrence of multiple lymphoid malignancies in the same patient remains rare [13, 14]. The incidence varies by age, sex, geography, and lymphoma subtype, and may be influenced by prolonged immunosuppression [15]. Similar to Richter’s transformation in chronic lymphocytic leukemia, transformation of indolent CTCL leads to aggressive clinical behavior, rapid deterioration, resistance to therapy, and poor survival, with reported median survival of 19–36 months [16].

The risk of developing a second lymphoma is markedly elevated—approximately 20-fold for Hodgkin lymphoma and more than fivefold for non-Hodgkin lymphoma. The underlying mechanisms remain unclear, but may involve prior MF therapies, immune defects, or genetic and environmental predisposition. Whether MF specifically predisposes to second T-cell lymphomas is still unknown, as registry data often classify subsequent events generically as non-Hodgkin lymphoma. Reports of MF progression into PTCL are limited [17], and most rely on morphology, immunohistochemistry, and conventional TCR analysis [18–20]. More comprehensive molecular studies are needed to clarify these events and to distinguish subclonal evolution from independent second malignancies.

In conclusion, we describe two patients with long-standing MF who developed lymph node and extranodal progression despite stable skin disease. Recognizing this atypical evolution requires clinical vigilance, careful clinicopathologic correlation, and access to high-throughput molecular sequencing.

Current CTCL diagnosis relies on clinical and histopathologic findings, supported by TCR gene analysis. Although increasingly used, NGS is not yet routine in T‑cell neoplasms. Our findings are in keeping with emerging evidence indicating that molecular data may refine diagnostic classification and prognostic assessment of CTCL, and disclose potential therapeutic targets [21–23]. Further integrated genomic studies such as NGS can help better define the patterns of CTCL progression.

## Data Availability

No datasets were generated or analysed during the current study.
